# Genetic sequence characterization and naturally acquired immune response to *Plasmodium vivax* Rhoptry Neck Protein 2 (PvRON2)

**DOI:** 10.1186/s12936-018-2543-7

**Published:** 2018-10-31

**Authors:** Najara C. Bittencourt, Juliana A. Leite, Ana Beatriz I. E. Silva, Tamirys S. Pimenta, João Luiz Silva-Filho, Gustavo C. Cassiano, Stefanie C. P. Lopes, Joao C. K. dos-Santos, Catarina Bourgard, Helder I. Nakaya, Ana Maria Revorêdo da Silva Ventura, Marcus V. G. Lacerda, Marcelo U. Ferreira, Ricardo L. D. Machado, Letusa Albrecht, Fabio T. M. Costa

**Affiliations:** 1 Laboratory of Tropical Diseases-Prof. Dr. Luiz Jacintho da Silva, Department of Genetics, Evolution, Microbiology and Immunology, University of Campinas-UNICAMP, Campinas, SP Brazil; 2 Instituto Carlos Chagas, Fundação Oswaldo Cruz – FIOCRUZ, Curitiba, PR Brazil; 3 Laboratório de Ensaios Clínicos e Imunogenética em Malária, Instituto Evandro Chagas/SVS/MS, Ananindeua, PA Brazil; 4 Instituto Leônidas & Maria Deane, Fundação Oswaldo Cruz – FIOCRUZ, Manaus, AM Brazil; 5 Fundação de Medicina Tropical-Dr. Heitor Vieira Dourado, Manaus, AM Brazil; 6 School of Pharmaceutical Sciences, University of São Paulo, São Paulo, Brazil; 7 Department of Parasitology, Institute of Biomedical Sciences, University of São Paulo-USP, São Paulo, Brazil

**Keywords:** Genetic diversity, RON2, *Plasmodium vivax*, Immunogenicity, Malaria

## Abstract

**Background:**

The genetic diversity of malaria antigens often results in allele variant-specific immunity, imposing a great challenge to vaccine development. Rhoptry Neck Protein 2 (PvRON2) is a blood-stage antigen that plays a key role during the erythrocyte invasion of *Plasmodium vivax*. This study investigates the genetic diversity of PvRON2 and the naturally acquired immune response to *P. vivax* isolates.

**Results:**

Here, the genetic diversity of PvRON2_1828–2080_ and the naturally acquired humoral immune response against PvRON2_1828–2080_ in infected and non-infected individuals from a vivax malaria endemic area in Brazil was reported. The diversity analysis of PvRON2_1828–2080_ revealed that the protein is conserved in isolates in Brazil and worldwide. A total of 18 (19%) patients had IgG antibodies to PvRON2_1828–2080_. Additionally, the analysis of the antibody response in individuals who were not acutely infected with malaria, but had been infected with malaria in the past indicated that 32 patients (33%) exhibited an IgG immune response against PvRON2.

**Conclusions:**

PvRON2 was conserved among the studied isolates. The presence of naturally acquired antibodies to this protein in the absence of the disease suggests that PvRON2 induces a long-term antibody response. These results indicate that PvRON2 is a potential malaria vaccine candidate.

**Electronic supplementary material:**

The online version of this article (10.1186/s12936-018-2543-7) contains supplementary material, which is available to authorized users.

## Background

Clinical manifestations of human malaria occur during the blood stage of infection by the *Plasmodium* parasite. Merozoites invade red blood cells in a process that involves specific interactions between parasite ligands and host cell receptors; the merozoites are propelled by actin/myosin motors through the moving junction (MJ). The MJ is a protein complex formed by Apical Membrane Antigen 1 (AMA1) and Rhoptry Neck Proteins (RON) 2, 4, 5 [1]. RON2 homologs are present in various species of the Apicomplexa phylum [1–4]. In *Plasmodium* vivax and *Plasmodium falciparum,* this protein is expressed in schizonts and secreted by the rhoptries at the end of the erythrocytic cycle [3]. Although the interaction between RON2 and AMA1 is essential to the invasion process [5–7], the mechanism of interaction is not well understood. RON2 is transferred to the red blood cell (RBC) membrane and adopts a surface-exposed loop that binds to a hydrophobic groove in AMA1, which is secreted by micronemes at the parasite surface [1, 7, 8]. This interaction triggers the junction formation and the invasion process. In the same microenvironment of erythrocyte invasion, anti-parasite acquired immunity occurs largely through the recognition of blood stage antigens expressed by the merozoite.

Thus, merozoite proteins are important targets and promising candidates for a malaria vaccine [9–12]. However, the development of a vivax malaria vaccines is still at an initial stage, and blood stage antigens that could be novel vaccine candidates are not well known [13]. RON2 is present in various *Plasmodium* species and is likely exposed to the host immune system during erythrocyte invasion, making this protein a potential target for antibody-mediated protective immunity and vaccine development.

The genetic diversity of a candidate antigen becomes relevant when pursuing an efficient protective immune response. The expression of *P. vivax* proteins with a high degree of polymorphism and the corresponding strain-specific immune response represent major obstacles to vaccine development [14, 15]. The high antigenic diversity of the parasite explains the slow development of naturally acquired immunity [16]. Thus, repeated antigen exposure over several years is necessary to generate a great repertoire of antibodies against different serotypes in an endemic area [17].

In malaria endemic regions, individuals are naturally exposed to malaria, and therefore, they produce specific immune responses against several strains. The acquired immunogenicity is generally short-lived, strain-specific and developed gradually after repeated infections [17–19]. This immunity can restrain parasitaemia, protecting the individual against severe disease and decreasing the risk of mortality.

In this work, the polymorphism patterns of *P. vivax* RON2 and the naturally acquired antibody responses to this antigen were characterized genetic diversity analysis and immunogenicity to PvRON2 lays a foundation for the potential future design and development of an effective PvRON2-based malaria vaccine.

## Methods

### Study area, population and sample collection

**Fig. 1:**
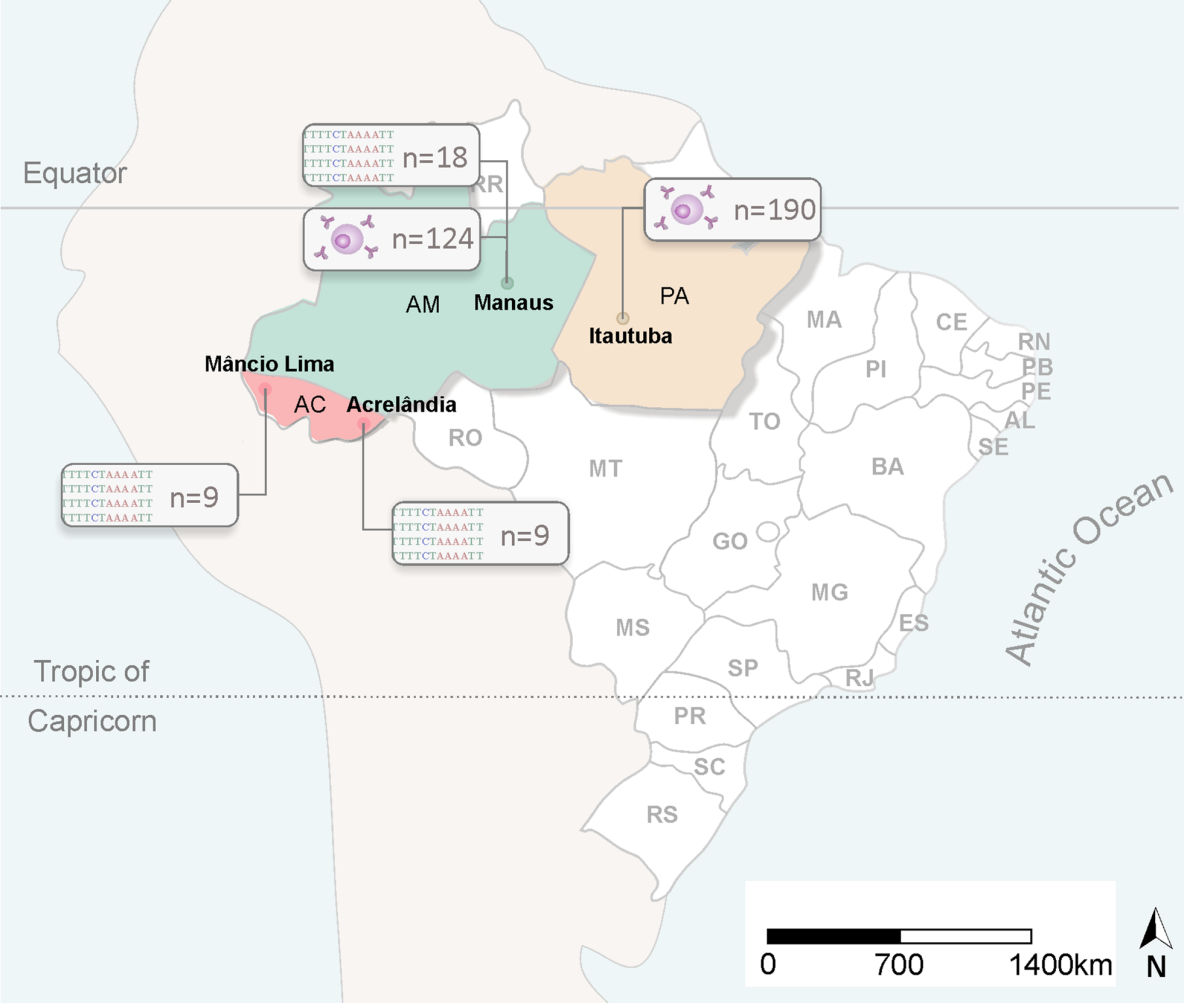
Geographic areas where *P. vivax* samples were collected. Samples used in the genetic diversity analysis were collected in Manaus, Amazon state and Mâncio Lima and Acrelândia, Acre state. Samples from Manaus and Itaituba, Pará state were used in the immunogenicity analysis. The number of samples collected at each location is indicated on the map

To evaluate the genetic diversity of *pvron2* (sequence from nucleotide 5481–6240 from the *pvron2* gene), a total of 36 *P. vivax* isolates were collected from patients in Manaus, Amazonas-Brazil at *Fundação de Medicina Tropical Dr. Heitor Vieira Dourado* (FMT-HVD) (CAAE-0044.0.114.000-11/CAAE 54234216.1.0000.0005) and in Mâncio Lima and Acrelândia, Acre State (Fig. 1) between 2011 and 2013 and in 2015 (936/CEP, 2010 and 1169/CEPSH, 2014).

Humoral immune responses to a recombinant PvRON2 (rPvRON2) antigen in Manaus (− 03°06′26″S; 60°01′34″W) (CAAE-0044.0.114.000-11/CAAE 54234216.1.0000.0005) and in Itaituba (04°16′34″S; 55°59′01″W), a gold mining area located in the most southwestern part of the State of Pará, were assessed (Fig. 1). The Annual Parasite Incidence designates Itaituba as an area with a high risk of malaria transmission (102.0 cases/1000 inhabitants per year) (CAAE—001.219.346-15). In contrast, Manaus presents a low malaria transmission risk (5.8 cases/1000 inhabitants per year).

Samples of peripheral blood were collected from the following three groups: (1) individuals acutely infected with *P. vivax* in Itaituba (n = 93); (2) individuals who were not infected but previously had malaria (n = 97); and (3) acutely infected individuals from Manaus (n = 124). All individuals were previously diagnosed by thick blood film microscopy screens, and *P. vivax* mono infection was also determined by nested PCR, as previously described [20].

### Target sequence

The PvRON2 gene has a length of 6612 bp. A synthetic gene fragment was designed based on the nucleotide sequence of the *pvron2* Sal1 strain (PlasmoDB PVX_117880). This sequence is only partially located in the PvRON2 region that binds to AMA1 during the MJ formation. Nevertheless, the selected region presented a high score in the antigenicity analysis, according to the Kolaskar and Tongaonkar method, using the program Immune Epitope database and Analyses Resource (IEDB Analysis Resource) [21].

### Amplification and sequencing of pv*ron2* from Amazonian isolates

Genomic DNA from *P. vivax* isolates collected in Manaus was extracted using standard phenol–chloroform methods [22]. The DNA templates from the isolates collected in Mâncio Lima and Acrelândia were isolated from 200 µL of whole blood using QIAamp DNA blood kits (Qiagen, Hilden, Germany), with a final DNA elution volume of 200 µL, according to the manufacturer’s instructions. DNA samples were stored at − 20 °C prior to use.

DNA samples of *P. vivax* were used as the template for the amplification of the *pvron2* 759pb sequence. Three DNA fragments were PCR-amplified to obtain the complete fragment. The oligonucleotide sequences used in this study are listed in Additional file 1. The PCR conditions used to amplify fragments one and three were as follows: 1 cycle of 5 min at 95 °C followed by 35 cycles of 30 s at 95 °C, 45 s at 60 °C, 1 min at 72 °C and a final cycle of 5 min at 72 °C. To amplify fragment two, the PCR conditions were as follows: 1 cycle of 5 min at 94 °C followed by 35 cycles of 30 s at 94 °C, 30 s at 60 °C, 45 s at 72 °C and a final cycle of 5 min at 72 °C. The reactions were performed using a reaction mixture containing 2.5 mM MgCl_2_, 0.5 mM each dNTP (Invitrogen), 0.5 units of *Platinum* Taq polymerase (Invitrogen) and 1 µM of each oligonucleotide primer in a final volume of 50 µL. The purified PCR product was sequenced using 3730xl DNA Analyzer (Applied Biosystems).

### Sequence alignment and analysis

Amplified *pvron2* sequences from 36 Brazilian Amazon isolates were analysed. The fragments were assembled using CodonCode Aligner v. 6.0.2, and the sequence data was deposited in the GenBank (accession numbers are listed in Additional file 2). Single nucleotide polymorphisms (SNPs) were identified in the alignment of 103 pv*ron2* sequences from 7 other countries (Thailand, Mexico, Mauritania, China, Peru, Colombia and North Korea), which were previously deposited in the PlasmoDB [23], and GenBank [24] databases. All sequences were compared to the *P. vivax* reference sequence Sal-1 strain (PlasmoDB: PVX117880) using CLC Sequence Viewer 7.

### Expression, purification and confirmation of the PvRON2 protein in *Escherichia coli*

A PvRON2 gene fragment encoding amino acid residues 1828–2080 was obtained from the genomic DNA of the *P. vivax* Sal I strain. This fragment was codon optimized for *E. coli* and subsequently cloned into the pGEX 4T-1 vector (synthetized by GenScript USA Inc. Piscataway, New Jersey). For recombinant GST-PvRON2 protein expression, the pGEX4T-1_*pvron2* plasmid was transformed into competent STAR BL21(DE3) *E. coli* cells by heat shock [25]. Then, the bacteria cultures were grown according to the manufacturer’s instructions. Briefly, 100 mL of overnight culture was transferred into 3 L of LB containing ampicillin (100 µg/mL) and incubated at 37 °C with shaking. When the culture reached an OD_600_ of 0.6, protein expression was induced by the addition of 0.8 mM IPTG for 4 h at 37 °C. The culture was pelleted by centrifugation (6000×*g*, 10 min), resuspended in 25 mL of lysis buffer (10 mM Tris HCl pH 8.0, 150 mM NaCl, 1 mM EDTA) in the presence of 1× Complete Protease Inhibitor Cocktail (Roche, Mannheim, Germany) and incubated for 1 h on ice. The sample was then disrupted using an M-110 L Pneumatic High Shear Fluid Processor (Microfluidcs). Next, cell fragments were pelleted by 30 min of centrifugation at 10,000×*g*. Finally, the supernatant was collected and analysed using SDS-PAGE and Western Blot to confirm protein expression.

### Measurement of antibody reactivity to rPvRON2

Naturally, acquired IgG and IgM antibodies against rPvRON2 were measured in the plasma samples by direct enzyme-linked immunosorbent assay (ELISA). Plasma samples from infected (n = 93) and non-infected (n = 97) individuals from Itaituba, and infected individuals from Manaus (n = 124) were evaluated for the presence of IgG antibodies to rPvRON2. The same groups were evaluated for the presence of IgM antibodies to rPvRON2, as follows: infected individuals from Itaituba (n = 56), non-infected individuals from Itaituba (n = 97), and infected individuals from Manaus (n = 68). Samples from non-infected individuals from a non-endemic region were used as negative controls (n = 21).

High-protein binding 96-well ELISA plates were coated with 50 μL of rPvRON2 at 5 μg/mL in 0.05 M carbonate-bicarbonate, pH 9.6, overnight at 4 °C. Then, plasma samples (100 µL) diluted 1:100 were added to each well and incubated for 1 h at room temperature. For the detection of bound antibodies, the samples were incubated with a 1:2000 dilution of peroxidase-conjugated goat anti-human IgG or IgM (Sigma). The optical density (OD) was measured at 490 nm using CLARIOstar data analysis.

To avoid a bias in the results caused by the possible reactivity of the GST tag during protein expression, the excess band size was measured on the SDS PAGE gel using ImageJ. To calculate the protein surplus, plates were coated with GST. The values obtained in each sample reaction to GST were subtracted from the value obtained in the reaction against PvRON2.

All plates tested were normalized using the values of the anti-GST controls (4 well per plate). The cutoff value was calculated as the mean plus three standard deviations of the negative control. The reactivity indices (RIs) were obtained from the ratio of the absorbance values of each sample and the cutoff value. The prevalence of IgG and IgM against the rPvRON2 antigen was considered positive if the (RI) values were higher than 1.0.

The detection of IgG subclasses was performed as mentioned above, except that IgG1 (HRP), IgG2 (HRP), IgG3 (HRP) and IgG4 (HRP) specific secondary monoclonal mouse anti-human antibodies (abcam) diluted 1:2000 were used. The results were expressed as the RI ± SEM (standard error of the mean).

### Measuring cytokine levels

The plasma levels of the cytokines IL-6, IL-10, IFN-γ and TNF were quantified by flow cytometry using the BD IL-6, IL-10, IFN-γ, TNF Human Flex Set (BD Bioscience Pharmingen, San Diego, Ca, USA) following the instructions provided by the manufacturer. Data analyses were performed using the FACSDiva software (BD Biosciences, San Jose, CA, USA). The cytokine concentrations in each sample were determined based upon standard curves. The plasma cytokine concentrations for each sample were extrapolated from the standard curves, and the data were expressed as ρg/mL.

### Correlation coefficients and network analysis

As the antibody levels were not normally distributed, nonparametric tests were used. Spearman’s correlation was applied to assess the association between antibody levels with the following parameters: age, parasitaemia, platelets, RBC, haematocrit, haemoglobin, IL-6, IL-2, IL-10, IL-4, TNF and IFN-γ. Correlation networks were generated by the analysis of relationships among each mediator measured in the plasma samples. The systemic levels of each mediator were input in the R software (v. 3.4.3). Initially, pairwise Spearman’s correlation coefficients were calculated using the R programming language. Along with the Spearman rank-order correlation coefficient, the p value to test for non-correlation was evaluated using p ≤ 0.05 as a cutoff. Moreover, based on the Spearman correlation coefficient, the same software was applied to identify links (edges) of interaction between the mediators (nodes). The correlation strength is represented by edge transparency and width; positive correlations are represented by red edges, and negatives correlations are represented by blue edges. Following this approach, each mediator was selected as a target, and the R software was used to perform a search within the other mediators for those that were associated with the target, in terms of correlation strength. As a result, the features related to the selected target were linked. This process was repeated for each mediator, and the result was the inferred network among the input values. To analyse the structure of the networks; the graphics of the network analysis were customized using Cytoscape software (v 3.5.1). The prefuse force-directed layout was applied, which, in the equilibrium states for the system of forces, represents the correlation strength, the edges with uniform length, and nodes that are not connected by an edge tend to be drawn further apart.

### Statistical analysis

Fisher’s test was applied to compare the prevalence of positive responses, and differences between medians and cytokine levels were assessed with the Mann–Whitney U-test using GraphPad Prism. A p-value of p < 0.05 was considered significant.

## Results

### PvRON*2* is a conserved antigen

A total of 36 pv*ron2* sequences corresponding to the amino acid residues 1828–2080, based on the *P. vivax* reference sequence Sal-1 (PlasmoDB PVX_117880), were generated from gDNA samples of *P. vivax*-infected individuals from Manaus, Mâncio Lima and Acrelândia in the Amazon region (Fig. 1). The Brazilian sequences were then compared with 103 other sequences from eight countries that were previously deposited in GenBank and PlasmoDB (Additional file 3).

Sequence analysis revealed that pv*ron2*_5482-6240nt_ is highly conserved among *P. vivax* isolates worldwide. Only two synonymous nucleotide substitutions were found among 118 sequences. One of these substitutions was observed at position 76 nt and was detected in 47 isolates from six out of the nine countries analysed. Another substitution was found at position 228 nt in one sequence from Peru (Additional file 3B). Non-synonymous substitutions were not detected in any of the analysed sequences, indicating that *pvron2* in a highly-conserved protein (Additional file 3A).

### Demographic profiles of individuals from Itaituba

In this study, a total of 93 infected and 97 non-infected individuals from Itaituba were analysed. A higher frequency of infected individuals was observed among males (80.6%) compared with females (19.4%). The median age of infected individuals was 32 years old, which was significantly lower than that of uninfected individuals (43 years old). In the non-infected group, the majority of individuals reported suffering more previous episodes of malaria compared to the infected group of patients. Malaria infection significantly affected the prevalence of anaemia (24.7% vs 9.3% for the infected and non-infected groups, respectively) and thrombocytopaenia (79.6% vs 19.6 for the infected and non-infected groups, respectively) (Table 1).

**Table 1:** Epidemiological parameters of the subjects exposed to malaria in the gold mining region (Itaituba)

Characteristics	Infected (93)	Non-infected (97)	p*
Gender, male (%)	80.6	62.4	0.001
Age, median years (range)	32	43	< 0.0001
Past malaria infections			
≥ 4	41.6	74.2	< 0.0001
< 4	58.4	25.8	
Haemoglobin (g/dL)	14.0	14.3	
Anaemia (%)	24.7	9.3	0.008
Platelets (cells/mm^3^)	104,000	190,000	
Thrombocytopaenic (%)	79.6	19.6	< 0.0001
Cytokines (pg/mL)			
INF-γ	2.04	0	0.0002
IL-6	24.38	3.02	< 0.0001
IL-10	81.95	0.22	< 0.0001
IL-2	0	0	0.09
IL-4	0	0	0.63
TNF-α	3.09	0.86	0.03

Anaemia was considered positive when haemoglobin levels were under 13 g/dL for men or under 12 g/dL for women

Thrombocytopaenia was defined as a platelet count of less than 150 × 10^3^ per µL

* p-values were calculated from the Chi-squared test for qualitative variables or the Mann–Whitney test for non-parametric continuous variables

### Naturally acquired antibody response towards rPvRON2

Recombinant PvRON2 was successfully expressed in *E. coli* STAR cells as a GST fusion protein. The purity and quality of the recombinant protein were verified by SDS-PAGE, which revealed a single band of approximately 56 kDa. The generated recombinant protein (rPvRON2) was recognized by the plasma from *P. vivax*-infected patients, confirming its immunoreactivity (Additional file 4).

To evaluate PvRON2 as a vaccine candidate, the immune response towards this antigen was assessed. A total of 190 individuals from Itaituba (93 infected and 97 non-infected) were evaluated for the presence of a naturally acquired IgG antibody response to rPvRON2, and 153 individuals (56 infected, 97 non-infected) were evaluated for an IgM response. In addition, 124 and 68 infected patients from Manaus were also analysed for IgG and IgM responses, respectively, to evaluate possible regional differences among infected patients.

**Fig. 2:**
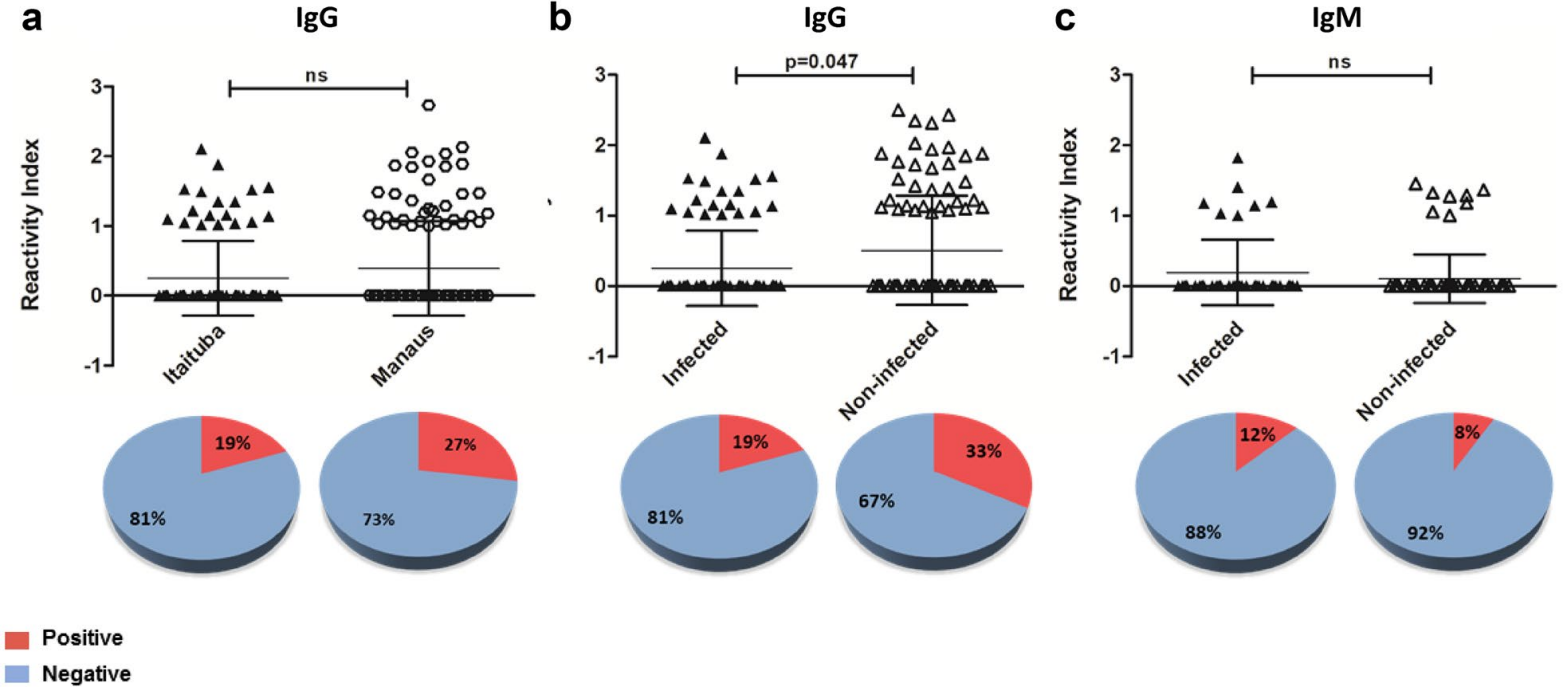
Human IgG and IgM antibody responses to rPvRON2. Reactivity indices of **a** IgG per person against rPvRON2 in infected individuals from Itaituba (n = 93) and infected individuals from Manaus (n = 124), **b** IgG response from infected individuals from Itaituba (n = 93) and non-infected individuals who had malaria in the past (n = 97), **c** IgM response from infected individuals (n = 56) and non-infected individuals (n = 97) from Itaituba, measured by ELISA. The differences in the total number of individuals evaluated for each protein are due to samples running out of plasma. Error bars indicate the mean with standard deviation. p values are indicated in the figure. *ns* not significant. Significant differences were calculated by Fisher’s test

IgG antibodies naturally acquired were prevalent in 19% (18/93) of the samples from infected individuals from Itaituba. The reactivity indices of anti-PvRON2 IgG antibodies were similar between infected patients from Itaituba and Manaus. While the individuals from Itaituba presented a prevalence of 19%, individuals from Manaus showed a prevalence of 27% (34/124) (Fig. 2a). Finally, the highest level of IgG response was found in the non-infected individuals from Itaituba (Fig. 2b), with a prevalence of 33% (32/97). The presence of naturally acquired antibodies towards rPvRON2 in plasma samples from Itaituba in individuals who had been infected with malaria in the past but exhibited no parasitaemia at the moment of blood harvest was significantly higher compared with infected individuals (p = 0.047), suggesting a possible maintenance of the PvRON2-specific antibody response.

**Fig. 3:**
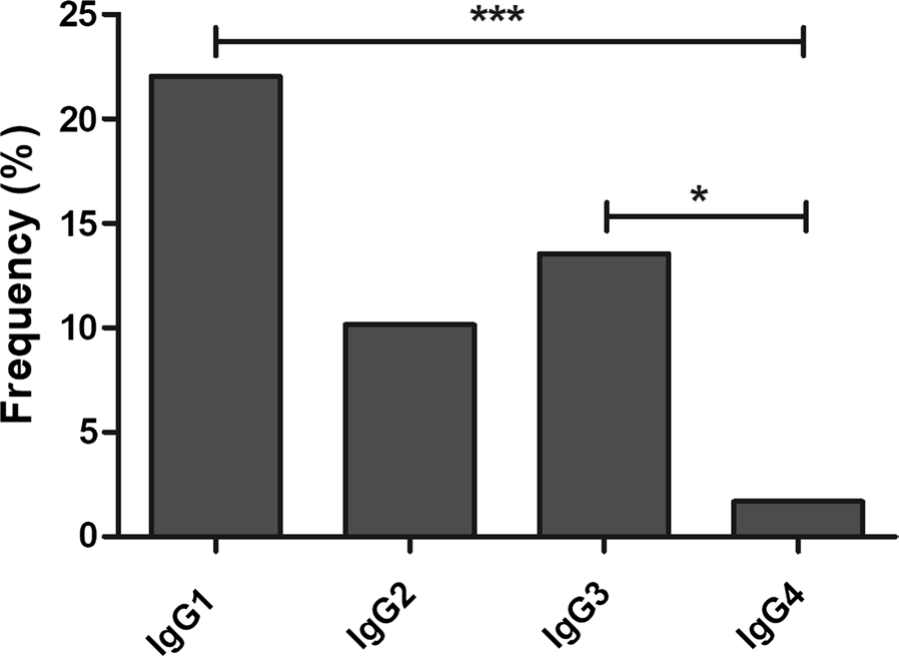
Frequency of IgG subclasses. Prevalence of IgG1, IgG2, IgG3 and IgG4 antibodies against PvRON2 in IgG-positive malaria individuals (n = 59). The differences in the total number of individuals evaluated corresponded to samples that lacked plasma. *p < 0.005 **p < 0.0001. The Fisher’s test was used to analyse differences between each IgG subclass response

Some of the individuals with a positive reactivity index, both from Manaus and Itaituba (n = 59), were isotyped to detect IgG subclasses (IgG, IgG2, IgG3, IgG4). The results revealed that 22.03% of individuals exhibited anti-IgG1, 10.16% exhibited anti-IgG2, 13.55% exhibited anti-IgG3 and only 1.69% exhibited anti-IgG4 antibodies (Fig. 3).

The prevalence of IgM antibodies was lower compared to IgG, as follows: 12.5% (7/56) for patients from Itaituba and 8% (8/97) for non-infected individuals from Itaituba (Fig. 2c). In samples from Manaus, only 4% (3/68) of individuals were positive for an IgM antibody response to rPvRON2.

### Cytokine levels and network analysis

**Fig. 4:**
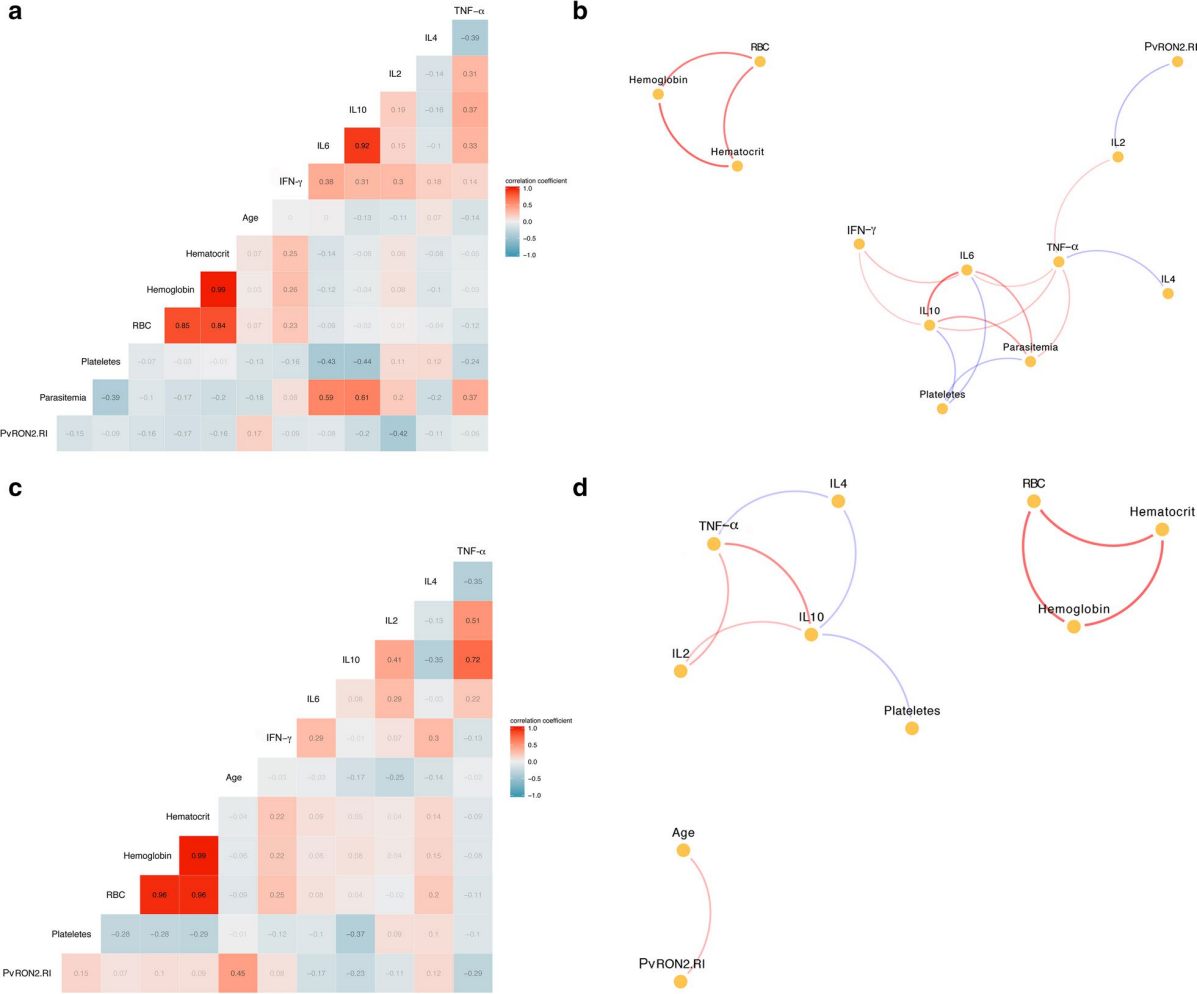
Multivariate correlation coefficients and networks in infected and non-infected individuals from Itaituba. Spearman’s correlation was applied to assess the association between the PvRON2 reactivity index with age, parasitaemia, platelets, RBC, hematocrit, hemoglobin, IL-6, IL-2, IL-10, IL-4, TNF-α and IFN-γ, and correlation networks were generated by the analysis of the relationship among each mediator measured in the plasma samples in **a**, **b** the infected group and **c**, **d** the non-infected group. Each connecting line (edge) represents a significant interaction (p < 0.05) detected by the network analysis using the R software. Correlation strength is represented by the tile or edge color transparency and width. Positive correlations are represented with red tiles/edges, and negatives correlations are represented with by blue tiles/edges

To evaluate a possible correlation between the levels of cytokines and several parameters, the plasma levels of TNF, IFN-γ, IL-2, IL-4 IL-6 and IL-10 in all individuals from Itaituba were measured (Table 1). The cytokine levels were found to be positively associated with age, parasitaemia, platelets, RBC, haematocrit and haemoglobin; one significantly negative correlation was detected between rPvRON2 reactivity index (RI) and IL-2 (p < 0.05; r = − 0.58) in infected individuals (Fig. 4a, b). No significant correlations were observed between the rPvRON2 reactivity index and the plasma levels of TNF, IFN-γ, IL-6, or IL-10 when analysed in both the non-infected and infected groups. In addition, a positive significant correlation between RI and age was observed in non-infected individuals (Fig. 4c, d).

**Fig. 5:**
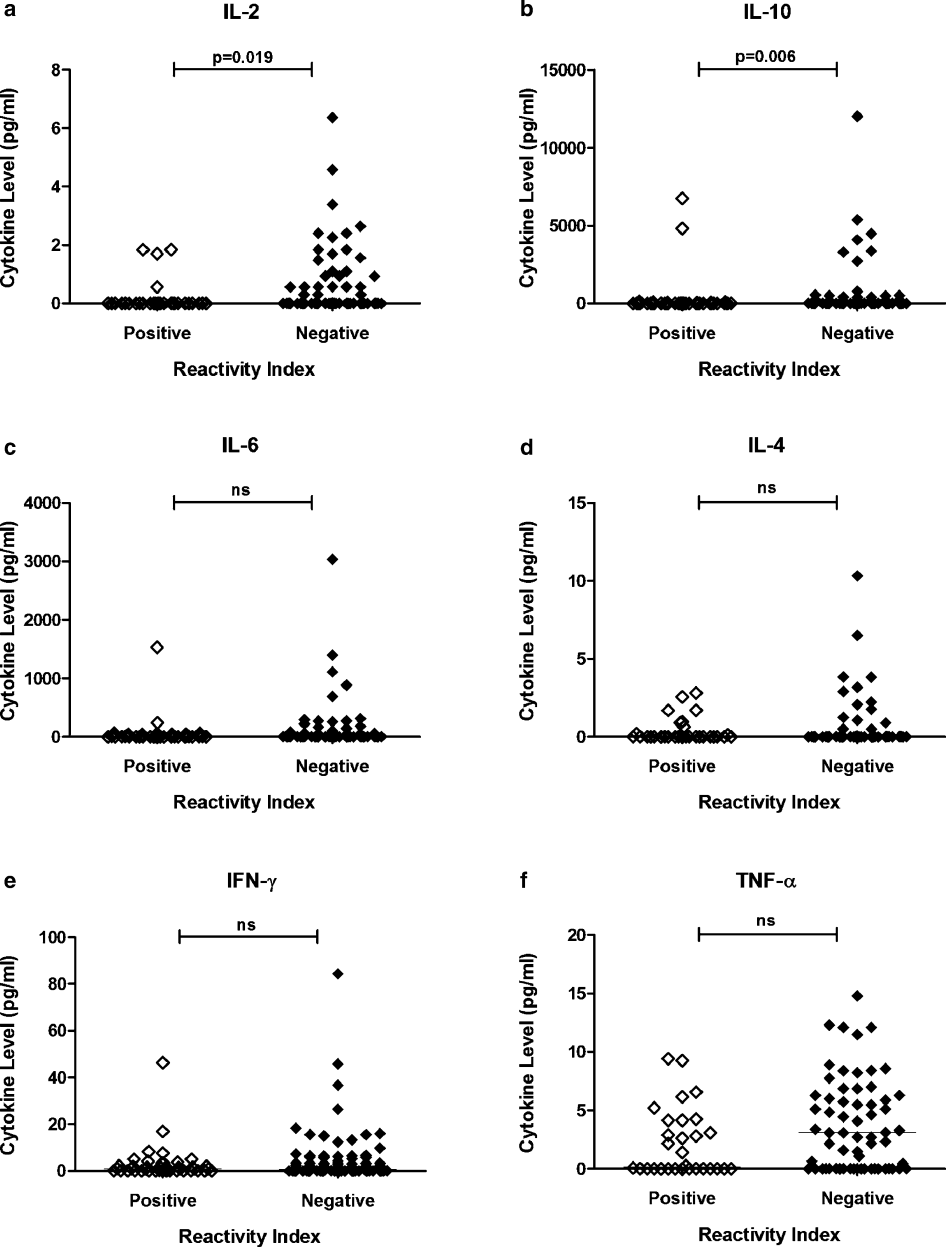
Cytokine Levels and the immune response to rPvRON2. Plasma samples from Itaituba were divided in two groups, with positive (RI > 1; n = 41) and negative (RI < 1; n = 103) reactivity indices against PvRON2. The levels of the cytokines: **a** IL-2, **b** IL-10, **c** IL-6, **d** IL-4, **e** INF-γ, **f** TNF-α were evaluated by flow cytometry. Bars indicate the median. p values are indicated in the figure Significant differences were calculated by the Mann–Whitney test. *ns* not significant

When cytokine levels were compared between individuals with a positive RI and a negative RI, a significant difference for both IL-2 (p = 0.019) and IL-10 (p = 0.006) was observed; individuals with anti-PvRON2 antibodies had lower levels of IL-2 and IL-10 when compared to individuals with a negative reactivity index (Fig. 5).

## Discussion

Many currently identified vaccine candidates present a high degree of polymorphism [26–28]. Selective pressure exerted by the host immune system, leads to the establishment of allele variants in the parasite population, reflected on the level of polymorphism rate [29]. One of the greatest challenges in the development of effective vaccines which are capable of generating an efficient antigen-specific immune response is the high level of polymorphic sequences. Genetic variability has been assessed in many important *P. vivax* vaccine candidates, such as CSP [30, 31], DBP [32, 33], MSP-1 [33, 34], MSP-3 alpha [35], MSP-4 [36], MSP-5 [37], MSP-7 [38], MSP-9 [39], RAP-1 [40], RAP-2 [40], Pvs48/45 [41], PvGCS1 [42], TRAP [43], and PvCelTOS [44].

Proteins involved in the MJ complex formation have also been characterized. PvAMA1 has been analysed and characterized in multiple studies on field isolates from different endemic area [29, 33, 45, 46]. PvAMA1 is a highly polymorphic antigen [29, 45, 46]. The complete AMA1 gene contains an ectodomain with three distinct subdomains (DI, DII and DIII) separated by disulfide bonds between the cysteine residues [47]. DI exhibits a higher mutation ratio and level of diversifying selection in *P. falciparum* [48, 49], whereas most of the polymorphic sites of AMA1 occur in domains I and II in *P. vivax* isolates  [46, 50, 51].

PfRON2 and PvRON2 interact with a hydrophobic groove in AMA1 [52, 53]. It has been recently reported that PvRON2-RI (957–1288 AA) [52–54] and PvRON2-RII (1850–2085 AA) [54] bind to PvAMA1 DI [52, 54] with high affinity [54], and in both *P. falciparum* and *P. vivax* species it was observed that the residue Tyr251, which was reported to be essential for RON2 binding [7, 55], is conserved [46, 55].

Among the RON proteins belonging to the invasion complex, PvRON4 and PvRON2 have been characterized regarding polymorphisms [56–58]. PvRON4 is conserved, with a low number of SNPs. However, there exist many haplotypes, due to the presence of tandem repeats in the N-terminal region. However, the central and C-terminal regions are highly conserved, likely because they are under functional constraint [58].

Certain pv*ron2* sequences described exhibit a high level of conservation in specific regions [56, 57]. Here, a fragment corresponding to 5482–6240 nt, which encodes PvRON21828-2080AA was analysed. This region was chosen based on the following two characteristics: its functional importance to the MJ formation during invasion, given that this region is partially located in the RII region of PvRON2, and its antigenicity, which was predicted using the IEDB Analysis Resource.

Polymorphisms in PvRON2_1828–2080_ were identified in samples collected from individuals infected with malaria from three different regions of the Brazilian Amazon, and these samples were compared with isolates from other countries. The alignment of 36 nucleotide sequences from the Brazilian isolates revealed only one SNP, and when these sequences were compared with other sequences deposited in PlasmoDB and GenBank, two SNPs were identified with no amino acid changes. The high degree of conservation of this sequence is likely due to the functional role of the interaction between AMA1 and RON2 during the MJ formation [6, 7].

In addition to evaluating the genetic variation of antigens, analysis of the natural immune response is essential for vaccine development. Given the importance of PvRON2 during the invasion process, which makes this protein a potential target of the immune system, in this study was analysed the prevalence of antibodies from infected individuals from two different endemic areas in the Brazilian Amazon region (Itaituba, Pará State and Manaus, Amazonas State).

Samples from Itaituba were collected from mining regions with a high migration rate of workers who were often previously exposed to the malaria vector. Massive human influx and deforestation have greatly impacted the ecosystem, which has promoted a greater proliferation of mosquitoes, boosting the number of malaria cases [59–61]. In contrast, in the Manaus region, malaria transmission is characterized by the migration of people from rural to urban/peri-urban areas [61]. However, when the natural acquired humoral response was evaluated, there were no significant differences in the prevalence of PvRON2 IgG antibodies between the samples from the two regions. Furthermore, the analysis of IgG subclasses in a portion of the sample group (n = 59), including samples from both regions, revealed a significantly higher prevalence of IgG1 and IgG3. These antibodies are predominant in naturally acquired immune responses against other *Plasmodium* antigens in malaria endemic regions [62–64].

Subsequently, infected patients from Itaituba were compared with a group of non-infected individuals from the same region. Interestingly, this analysis revealed that non-infected individuals had a significantly higher IgG response, indicating a long-lasting immune response against the PvRON2 antigen. Because no precise information was available, it was not possible to correlate the reactivity index to the number of previous malaria exposures.

In non-infected individuals, a positive correlation between RI and age was observed. This type of association has been described for several *Plasmodium* antigens [65–68], suggesting age dependence and a possible maturation of the immune system over time. A lower prevalence of IgM compared to IgG was observed in all groups. Non-infected individuals had an even lower prevalence compared with the infected individuals from Itaituba, although this difference was not significant. Low reactivity was expected, as IgM is generally produced during the first weeks after antigen recognition and decreases over time after the first activation, whereas IgG responses can increase following subsequent exposures to the antigen, often due to the humoral immunological memory [69].

The only published work evaluating PvRON2 antigenicity analysed the natural acquired immune response against four in silico-predicted B-cell epitopes of PvRON2, none of which is located in the RI or RII functional regions. Although there was an immune response against these epitopes, the response was low [70].

Higher levels of IL-10 are positively associated with high parasitaemia [71, 72]. In the present study, individuals who exhibited a positive reactivity index against PvRON2 exhibited lower plasma levels of IL-10. However, this group consisted primarily of non-infected individuals who were previously infected with malaria (without parasitaemia). Thus, it is possible that low levels of this cytokine are associated with the low parasitaemia in this group and not directly associated with reactivity against PvRON2.

RON2 is a potential vaccine candidate, as blocking the interaction between AMA1 and RON2 inhibits erythrocyte invasion [6, 7]. Several studies have aimed to verify the efficacy of AMA1 as a vaccine candidate [11, 73–75]. However, PfAMA1 vaccines did not provide significant protection against malaria in clinical trials [75, 76]. Meanwhile, rats immunized with a PfAMA1-RON2L complex, produced qualitatively better *P. falciparum* inhibitory antibodies upon invasion of RBCs compared with IgG elicited by the formulation containing only PfAMA1 [77]. Vaccination with this complex provided significantly higher protection in mice [77] and *Aotus* monkeys [78] compared with a formulation with PfAMA1 alone. In addition, a study with another fragment of the PfRON2 sequence (84aa–968aa) demonstrated that IgG antibodies against this sequence are associated with clinical protection [79].

## Conclusions

Taken together, the findings in this study demonstrate that PvRON2_1828–2080_ is conserved and, moreover, there is a possible persistence of the immune response against this antigen. The data presented here suggest that PvRON2_1828–2080_ may be a potential candidate to overcome the antigenic diversity limitations in vaccine design in future studies.

## Additional files

**Additional file 1.** Primer sequences used for the amplification of *pvron2* (5482–6240 nt).

**Additional file 2.** Country, number of sequences, accession numbers and database used in the polymorphisms analyses.

**Additional file 3.** Multiple sequence alignment of *pvron2*. Alignment of representative *pvron2* sequences from Brazil and Peru, compared to the *P. vivax* Sal-1 strain (PlasmoDB PVX_117880). (A) Nucleotide sequences of region 5.482–6.240. (B) Amino acid sequences of region 1828–2080 aa. The alignment was made using VLC Sequence Viewer 7.

**Additional file 4.** Expression of recombinant PvRON2 (1828–2080 aa). Expression of rPvRON2 in bacterial expression system. (A) Expression of an ~ 56 kDa band corresponding to PvRON2 (1828–2080 aa) and the GST tag (~ 26 kDa). Lane 1: Molecular marker *Spectra Multicolor High Range Protein Ladder* (Thermo Scientific). Lane 2: rPvRON2. (B) Lane 1: Antibody recognition using plasma from malaria positive individual. Lane 2: Negative control.
