# Supplementary material for: Genetic sequence characterization and naturally acquired immune response to Plasmodium vivax Rhoptry Neck Protein 2 (PvRON2)
Source: Malar J. 2018 Oct 31;17:401. doi: 10.1186/s12936-018-2543-7 (PMC6208078; doi:10.1186/s12936-018-2543-7)
Supplement: Supplementary file 1 — Additional file 1. Primer sequences used for the amplification of pvron2 (5482–6240 nt). [file 12936_2018_2543_MOESM1_ESM.pdf]

Additional file 1. Primer sequences used for the amplification of *pvron2* (5482-6240 nt).

Fragment	Position (nt)*	Oligonucleotide	Sequence (5-3')
1	5.261-5.855	*pvron2*-F1	CAGAACGGTCAAGCCAAC
		pvron2-R1	CGATTTGGGTAGCAGCACAT
2	5.482-6.240	*pvron2*-F2	GCCTTCATCGAAATTGTAGACC
		pvron2-R2	CATGCAAGCTTGTGTGGAG
3	5.868-6.408	*pvron2*-F3	GAAGGCCATGAAAAATGGAG
		pvron2-R3	GGACAAAAATTTCCTCCACATG
