# Supplementary material for: Genetic sequence characterization and naturally acquired immune response to Plasmodium vivax Rhoptry Neck Protein 2 (PvRON2)
Source: Malar J. 2018 Oct 31;17:401. doi: 10.1186/s12936-018-2543-7 (PMC6208078; doi:10.1186/s12936-018-2543-7)
Supplement: Supplementary file 2 — Additional file 2. Country, number of sequences, accession numbers and database used in the polymorphisms analyses. [file 12936_2018_2543_MOESM2_ESM.pdf]

Additional file 2. Country, number of sequences, accession numbers and database used in polymorphisms analyses.

Country	nº of sequences	Access number	Data bank
Brazil	36	MF818108; MF818109; MF818110; MF818111; MF818112; MF818113; MF818114; MF818115; MF818116; MF818117; MF818118; MF818119; MF818120; MF818121; MF818122; MF818123; MF818124; MF818125; MF818126; MF818127; MF818128; MF818129; MF818130; MF818131; MF818132; MF818133; MF818134; MF818135; MF818136; MF818137; MF818138; MF818139; MF818140; MF818141; MF818142; MF818143	GenBank
Brazil	3	BrazilI; Brazil32; Belem	PlasmoDB
Thailand	13	Thailand_VKBT-100; Thailand_VKBT-101; Thailand_VKBT-71; Thailand_VKBT-72; Thailand_VKBT-94; Thailand_VKBT-95; Thailand_VKBT-98; Thailand_VKBT-99; Thailand_VKTS-36; Thailand_VKTS-37; Thailand_VKTS-39; Thailand_VKTS-45; Thailand_VKTS-52.	PlasmoDB
Peru	19	Peru00692; Peru00699; Peru06; Peru07; Peru08; Peru1008; Peru2025; Peru257; Peru259; Peru260; Peru262; Peru3133; Peru3136; Peru3232; Peru4023; Peru852; Peru858; Peru872; Peru99622.	PlasmoDB
China	20	BAM13385.1; BAM13384.1; BAM13383.1; BAM13382.1; BAM13381.1; BAM13380.1; BAM13379.1; BAM13378.1; BAM13377.1; BAM13376.1; BAM13375.1; BAM13374.1; BAM13373.1; BAM13372.1; BAM13371.1; BAM13370.1; BAM13369.1; BAM13368.1; BAM13367.1; BAM13366.1.	GenBank
China	6	China_NB-17; China_LZCH-13; China_NB-13-1; China_NB-15; China_NB-16; China_LZCH-20	PlasmoDB
Colombia	23	Columbia_30102100438-A; Columbia_30101099040; Columbia_30101099036; Columbia_30102100437; Columbia_30102100438-B; Columbia_30102100439; Columbia_30102100446; Columbia_30102100440; Columbia_30102100441-B; Columbia_30102100445;	PlasmoDB

		Columbia_30102100448; Columbia_30102100485; Columbia_30102100486; Columbia_30102100488; Columbia_30111110020; Columbia_30111110026; Columbia_30111110015; Columbia_30103103280; Columbia_30102100504; Columbia_30102100489; Columbia_30102100490; Columbia_30102100491	
India	2	IndiaNYC; IndiaVII	PlasmoDB
Mauritania	1	MauritaniaI	PlasmoDB
North Korea	1	NorthKorean	PlasmoDB
Mexico	15	Mexico_118-A; Mexico_161-04; Mexico_165-A; Mexico_203-04; Mexico_21-A; Mexico_267-A; Mexico_32-E-03; Mexico_330-A; Mexico_55-03; Mexico_566-A; Mexico_63-08; Mexico_760-A; Mexico_938-A; Mexico_980-A.; Mexico_1086-A	PlasmoDB
