# Supplementary figures and images for: Genetic sequence characterization and naturally acquired immune response to Plasmodium vivax Rhoptry Neck Protein 2 (PvRON2)
Source: Malar J. 2018 Oct 31;17:401. doi: 10.1186/s12936-018-2543-7 (PMC6208078; doi:10.1186/s12936-018-2543-7)

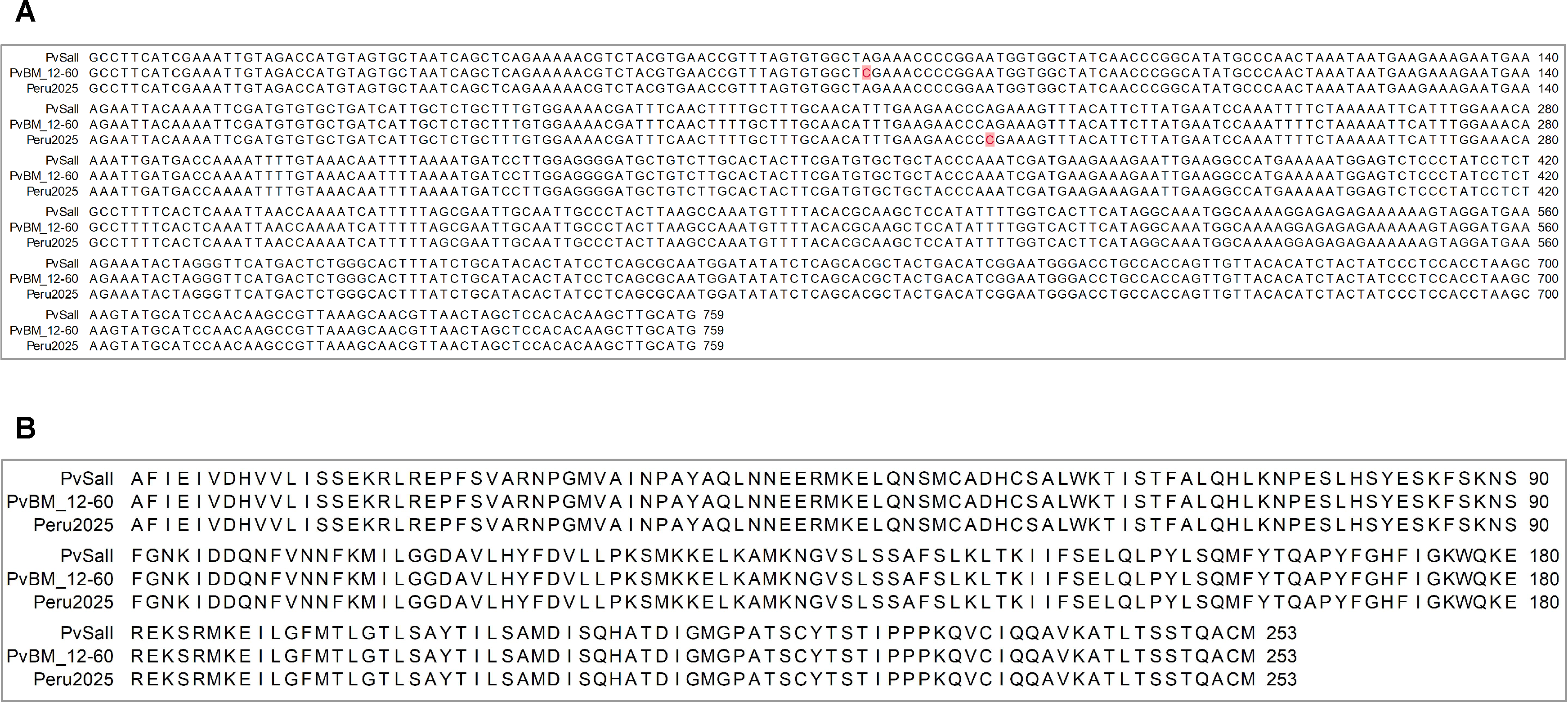

Supplement: Supplementary file 3 — Additional file 3. Multiple sequence alignment of pvron2. Alignment of representative pvron2 sequences from Brazil and Peru, compared to the P. vivax Sal-1 strain (PlasmoDB PVX_117880). (A) Nucleotide sequences of region 5.482–6.240. (B) Amino acid sequences of region 1828–2080 aa. The alignment was made using VLC Sequence Viewer 7. [file 12936_2018_2543_MOESM3_ESM.tif]

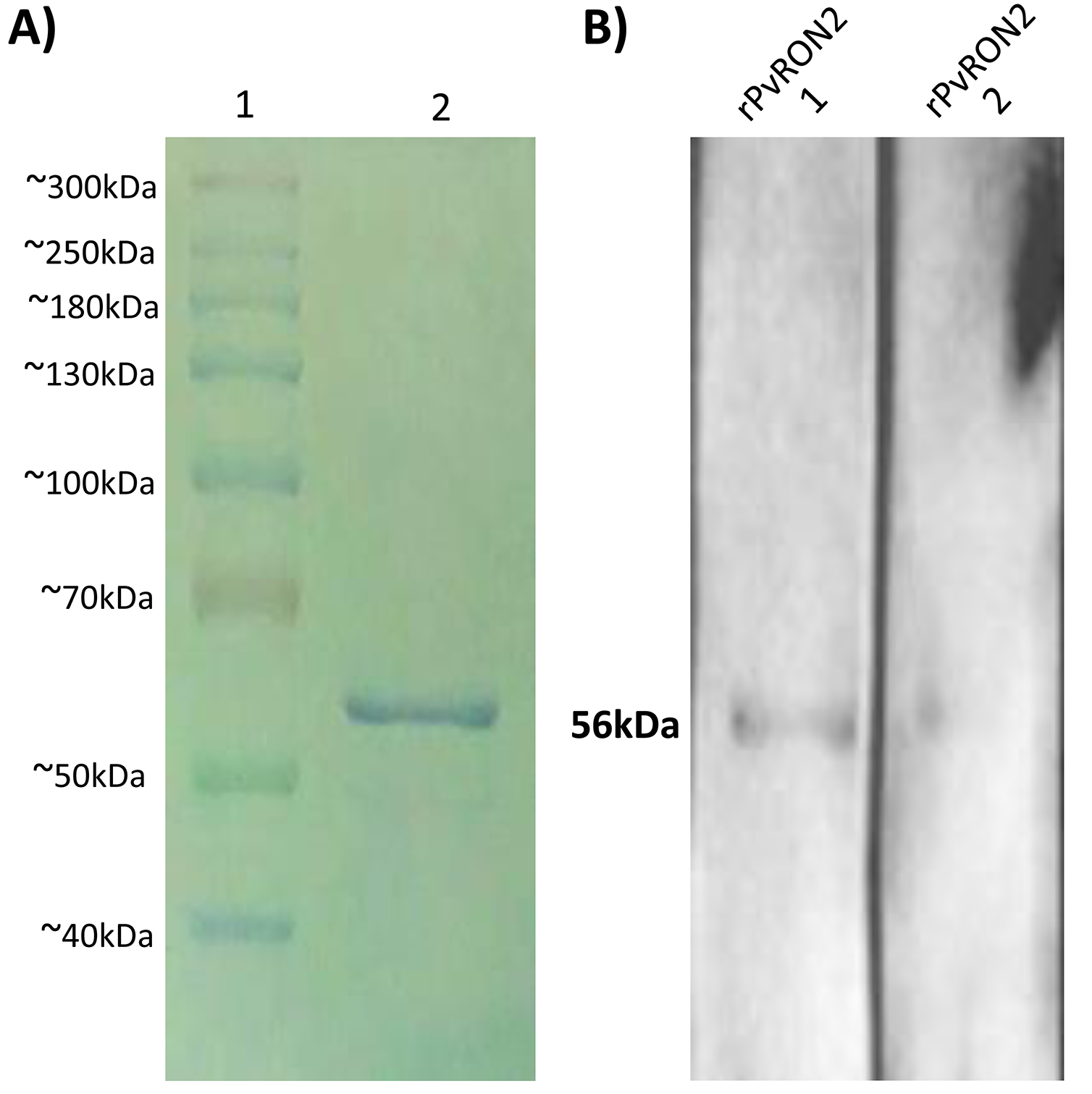

Supplement: Supplementary file 4 — Additional file 4. Expression of recombinant PvRON2 (1828–2080 aa). Expression of rPvRON2 in bacterial expression system. (A) Expression of an ~ 56 kDa band corresponding to PvRON2 (1828–2080 aa) and the GST tag (~ 26 kDa). Lane 1: Molecular marker Spectra Multicolor High Range Protein Ladder (Thermo Scientific). Lane 2: rPvRON2. (B) Lane 1: Antibody recognition using plasma from malaria positive individual. Lane 2: Negative control. [file 12936_2018_2543_MOESM4_ESM.tif]
